# The Book World of Medicine and Science

**Published:** 1911-08-26

**Authors:** 


### THE BOOK WORLD OF MEDICINE AND SCIENCE.

Crystals. By A. E. H. Tutton, D.Sc., M.A., F.R.S. The International Scientific Series. Volume XCVIII. (London: Kegan Paul, Trench, Trübner and Co., Ltd. 1911. Price 5s.)

Dr. Tutton has set himself the task of writing a popular account of the phenomena of crystallography, of which this fascinating little volume is the outcome. Unessential technical terms and forbidding mathematical formulæ have been rigidly excluded, with the result that the book can be read by the ordinary unscientific reader with interest and profit. The beautifully executed illustrations with which the text is interspersed cannot fail to open up to the tyro or amateur in crystallography a phase of the beauties of nature with which he was probably formerly entirely unacquainted. The author has banished any chance of his text proving tiresome by introducing historical matter relative to the pioneers in the science which he describes. In fact he has produced a treatise that is popular in the best sense of the word, and one which we can heartily recommend to those casually interested in scientific matters.

Yellow Fever and its Prevention. By Sir Rubert Boyce, M.B., F.R.S. (London: John Murray. 1911. Pp. 380. Price 10s. 6d. net.)

Last year we welcomed the admirable text-book of Ross on the Prevention of Malaria. This year it is the turn of yellow fever to be attacked, and it is matter for congratulation that the task was undertaken by one so well qualified to accomplish it as the late Sir Rubert Boyce. The practical researches which have established our modern system of yellow fever control are, of course, largely the work of the Americans, who abolished this scourge in Havannah and in Panama. The names of Guiteras and Gorgas will always remain associated with the magnificent work there accomplished, but where they showed the way there was a strange reluctance in many British colonies for medical officers to follow. That reluctance the author did much to overcome; his work in Barbados, West Africa, and many other tropical dependencies is too recent and too well known to need more than mention. It is very instructive to note from his pages how often colonial medical officers have been snubbed and intimidated by their civil official superiors and by the local Press for venturing to diagnose yellow fever and for doing their best to prevent its extension. Only last year, Sir Rubert shows, leading articles reflecting on local doctors, and crammed with an intolerant and ignorant fanaticism worthy of the Middle Ages at their worst, have been published by newspapers circulating in tropical countries. Into the different sections of treatment, prevention, history, epidemiology, and so forth, Sir R. Boyce enters fully. Space does not permit a detailed criticism of this exhaustive summary of the disease; suffice it to say that the volume is worthy of a place on the same shelf with that of Ronald Ross already alluded to. Speaking generally, the latter is the greater of the two, for it is a masterpiece; but this is no depreciation of the present volume, which is a most satisfactory and scientific résumé of the present state of knowledge of yellow fever, compiled by one who was really an expert on this subject.

The Medical Diseases of Children. By Reginald Miller, M.D., M.R.C.P. Illustrated. (Bristol: John Wright and Sons, Ltd. 1911. Pp. 541. Price 12s. 6d. net.)

The outstanding feature of this manual is one that will, we are convinced, meet with cordial appreciation—it is the practical usefulness of the book to students and general practitioners. We are well aware of the existence of one or two very excellent manuals on the diseases of children, but in welcoming Dr. Miller's addition to the text-books on this fascinating but difficult subject we realise that his book covers a wider area, and is at once both practical and yet of value as a work of reference. Taking the book as a whole we may sincerely congratulate the author on the serviceable nature of the result. When one man tries to get nearly the entire field of medicine within the limits of a moderate sized volume, it is unavoidable that faults or deficiencies should creep in, and it is impossible to compress large treatises on involved subjects into articles consonant with the purpose of a student's text-book. There is, indeed, but little to cavil at here, and the student who assimilates the teaching of Dr. Miller will find himself exceedingly well fortified. The arrangement of the book is very convenient, the illustrations excellent and numerous, and the many tables of summaries and differential diagnoses are sure to be found of great assistance. To single out sections for particular mention is an invidious task; the author himself is not aiming at monographic importance, but at all round up-to-date usefulness. Suffice it to say that such important matters as tuberculosis, rickets, the rheumatic infection and its allied diseases, and pyloric stenosis, are worthily dealt with. Certain affections whose course differs in no essential from the same disease in adult patients are not described in very great detail. The infective diseases are grouped according to their bacteriology, and the chief disorder is followed by accounts of the other manifestations of that particular bacterial infection—an arrangement at least theoretically sound and useful in emphasising the tendency to generalisation which is shown by such affections in children. On page 227 it is stated that when diphtheria complicates scarlet fever the result is usually fatal; this is far from being the case, and the statement requires amendment in a future edition. A slip occurs on page 513 in the paragraph about morphine, where the dilution is wrongly given. Not the least useful section of this book are the two Appendices, containing respectively dietetic and therapeutic measures and notes on the societies, etc., aiding invalid and defective children.

Prevention of Infectious Diseases. By Alvah H. Doty, M.D. (London and New York: D. Appleton and Co. 1911. Pp. 281. Price 10s. 6d.)

In virtue of his position as Medical Officer of Health to the port of New York the author of this book can command an attentive audience, and in this instance the lecture proves to be at once profitable and practical. Dr. Doty's views on the transmission of infectious disease by fomites are those of the majority of advanced sanitarians and are here discussed from a practical standpoint. He throws considerable doubt on the capability of fomites to retain infection in the active state, and though admitting the possibility under certain conditions that infection may be transmitted in this manner, he shows that greater importance must be attached to other factors, such as the unrecognised case, the carrier, and the part played by the insects. On the subject of marine hygiene we naturally get the benefit of the author's large practical experience, and also an insight into the working of the Health department of a large port, where international and personal interests have to be adjusted by the commonsense application of the principles of scientific sanitation. The chapter on plague is one of the most interesting in the book, and in it Dr. Doty sounds a note of warning against regarding as exclusive the view that plague is transmitted solely by the fleas infesting rats. A clear description is given of the actual details relating to disinfection, and the relative value and practicability of certain disinfectants, and here again the author strongly sets his face against certain procedures which have long been accepted, though probably useless and tending to overshadow the greater value of simpler methods of thorough cleanliness. The efficacy of boiling water is admirably emphasised. Reference is made to the author's own investigations on the use of a lime and sulphate of copper mixture as a cheap deodorant, and convincing details of its efficiency are given. There is also included an account of a temperature test on a large scale in which several thousands of temperatures were recorded in order to glean some reliable information as to the variations existing in healthy persons. The book is one that deserves a wide appreciation, and we regret to see that the price is one that we cannot but regard as excessive, considering its inexpensive format. Curiously enough the word "pathogenic" is used on pages 61 and 62, where "pathognomonic" is obviously meant.

Remarkable Comets. By William Thynne Lynn, B.A., F.R.A.S. Fifteenth edition, revised. (London: Samuel Bagster and Sons, Ltd. 1911. 6d. net.)

This unpretentious little volume gives a popular and interesting account of the comets, seen at various times in the world's history, of which trustworthy accounts have come down to us. No previous knowledge of astronomy is necessary before the reader can understand the author's meaning, and we feel sure that the booklet will continue to be as popular as it already has been.

A Manual of Bacteriology. By Robert Muir, M.D., and James Ritchie, M.D. (Henry Frowde, Oxford University Press, and Hodder and Stoughton, Warwick Square, E.C. 10s. 6d. net. 1910.)

Muir and Ritchie are so well known that this new edition, the fourth reprint of the book, needs no elucidative comment. The new edition is beautifully and accurately illustrated, and has been brought up to date so far as all but the rarest bacteriological conditions are concerned. It is the book for the intending D.P.H. student and for the laboratory worker who is preparing for examinations. Moreover, it is a volume that should be on the shelves of every practitioner's library.

Maternity Primer. By A. H. F. Barbour, M.D., LL.D. (Edinburgh: William Green and Sons. 1911. Price 1s. net.)

This small work is the outcome of a wish on the part of his pupils that the author's notes, at first privately printed for the use of nurses on commencing their maternity training, should be published. The teaching is accurate, though at times expressed in language which would seem more fitted for the intelligence of children than of grown women. The illustrations are admirable in their simplicity. The value of the book is enhanced by the inclusion of questions upon each paragraph into which the text is divided for convenience of reference.

Fifteenth Annual Report of the Lanark District Asylum, Hartwood, for 1909-10. (Glasgow: Robert Anderson. 1910.)

On May 31, 1910, there were resident in this asylum 912 patients, a decrease of 20 as compared with the same date in the previous year. A large proportion of aged men and women were admitted, of whom thirteen were over 50, twelve over 55, six over 60, seven over 70, and one over 80. This fact accounts to a large extent for the apparent increase of insanity in the district during recent years, since formerly many of these helpless senile persons would not have been sent to the asylum. The death-rate worked out at 8.3 per cent. on the average number resident, and at 6.4 on the total number under treatment. The recovery rate was 43.4 per cent. The maintenance rate is 8s. 9d. per week for district paupers, while that for out-district paupers and private patients is £32 per annum.

